# Influence of Polymorphisms in Innate Immunity Genes on Susceptibility to Invasive Aspergillosis after Stem Cell Transplantation

**DOI:** 10.1371/journal.pone.0018403

**Published:** 2011-04-04

**Authors:** Mark G. J. de Boer, Hetty Jolink, Constantijn J. M. Halkes, Pim L. J. van der Heiden, Dennis Kremer, J. H. Frederik Falkenburg, Esther van de Vosse, Jaap T. van Dissel

**Affiliations:** 1 Department of Infectious Diseases, Leiden University Medical Center, Leiden, The Netherlands; 2 Department of Hematology, Leiden University Medical Center, Leiden, The Netherlands; 3 Department of Molecular Epidemiology, Leiden University Medical Center, Leiden, The Netherlands; University of Leuven, Rega Institute, Belgium

## Abstract

The innate immune system plays a pivotal role in the primary defence against invasive fungal infection. Genetic variation in genes that regulate this response, initiated by pulmonary macrophages, may influence susceptibility to invasive aspergillosis in patients at risk. We investigated in a clinical setting whether common polymorphisms in Toll-like receptor (TLR) and cytokine genes involved in macrophage regulation are associated with susceptibility to invasive aspergillosis. Forty-four allogeneic stem cell transplantation recipients diagnosed with probable or proven IA according to the criteria of the European Organization for Research and Treatment of Cancer/Mycoses Study Group, were enrolled. The control group consisted of 64 allogeneic stem cell transplantation recipients without invasive aspergillosis. The *TLR4* 1063A>G single nucleotide polymorphism was associated with invasive aspergillosis when present in donors of allogeneic stem cell transplantation recipients (unadjusted OR 3.77 95%CI 1.08–13.2, p = 0.03). In a multivariate analysis, adjusted for occurrence of graft-versus-host-disease, Cytomegalovirus serostatus and duration of neutropenia, paired presence of the *TLR4* 1063A>G and *IFNG* 874T>A single nucleotide polymorphisms showed a trend towards increased susceptibility to invasive aspergillosis (p = 0.04). These findings point to the relevant immunological pathway involved in resistance to invasive aspergillosis and warrant further study of the effects of TLR and cytokine polymorphisms and their interaction, which may occur on different levels of the complex biological interplay between the immunocompromised host and *Aspergillus sp*.

## Introduction

It is incompletely understood why some stem cell transplant recipients develop invasive aspergillosis (IA), a cause of considerable morbidity and mortality, while others remain unaffected [1]. Clinical risk factors for the development of invasive fungal infections (IFI), have been identified, but such risks are not absolute [2], [3]. The host's or, in case of allogeneic stem cell transplantation (ASCT), the donor's genetic signature may influence susceptibility to acquiring manifest IA or at least affect its clinical course. Patients with an indication for treatment by an ASCT-procedure may have diverse hematological malignancies e.g. acute myeloid leukemia (AML), multiple myeloma (MM), chronic myeloid leukemia (CML), or other diseases. These hematological disorders are associated with an intrinsic risk of opportunistic infection, dependent on the stage and extent of the disease [4]. Furthermore, treatment related factors, most importantly graft-versus-host-disease (GVHD) and the duration of neutropenia, are known to influence the risk for development of IA [5], [6].

In-vitro- and animal studies indicated that the innate immune system plays a pivotal role in defence against IA by pathogen recognition and activation of appropriate host defence mechanisms in pulmonary macrophages [7], [8]. A family of pathogen recognition receptors (PRRs), the Toll-like receptors (TLRs), mediate this process through detection of fungal components and initiation of intracellular signalling pathways that lead to a pro-inflammatory cytokine response [9]–[13]. Only recently, a number of functional single nucleotide polymorphisms (SNPs) in *TLR4* as well as in *TLR1* and *TLR6* genes were associated with occurrence of IA in ASCT recipients [14]–[16].

However, the response of the innate immune system relies on a complex network of components which encompasses TLRs as well as molecules of signaling pathways (e.g. MyD88 and NFκB) and subsequently secreted cytokines [17]. Animal studies showed that depletion of IL-12 and IFN-γ delayed pulmonary clearance of *A. fumigatus* in mice [18]. Moreover, a high production of IL-12 and IFN-γ had a protective effect [19]. In humans, little is known about the role of these or other cytokines in the context of innate or acquired anti-fungal defense mechanisms and only scarce data is available to validate the clinical and experimental findings so far. Hence, we investigated the clinical relevance of common genetic polymorphisms in the TLR-mediated IL-12/IFN-γ loop to macrophage activation with regard to susceptibility to development of IA in ASCT recipients.

## Methods

### Study population

The study cohort consisted of 44 patients with hematological disorders and diagnosed with either proven or probable IA following ASCT according to the revised 2008 European Organization for Research and Treatment of Cancer and Mycosis Study Group (EORTC/MSG) criteria [20]. All patients were treated at the Leiden University Medical Center, a tertiary care and teaching hospital in the Netherlands. Patients were recruited from the database of the Department of Infectious Diseases. Sixty-four patients with comparable distribution of hematological disorders, but who did not develop IA, were enrolled in the control group. The control patients all received a allogeneic stem cell transplantation and were recruited for the study from the database of the Hematology department under the condition that DNA of the patient and the donor was available for study purposes. For control patients, the minimum follow-up time had to be 12 months. The ethnic background was Caucasian in both groups and all patients had undergone T-cell depleted ASCT. Demographic and clinical characteristics as well as outcome data were collected from the hospital's electronic database. The duration of neutropenia to the diagnosis of IA was defined as the number of consecutive days from the first day of a granulocyte count <0.5×10^6^ cells/L (determined ±3 times weekly) to the day that microbiological evidence of IA was first obtained. The study was endorsed by the local medical ethics committee. No standard prophylaxis active against *Aspergillus sp*. was used. Antifungal therapy was sometimes initiated on a pre-emptive basis, but always discontinued within 1 week if a probable or proven fungal infection was not diagnosed. Clinical characteristics per group are as summarized in table 1.

**Table 1:** Clinical characteristics of patients with underlying hematological disease with (cases) or without (controls) invasive aspergillosis after allogeneic stem cell transplantation.

Variable	ASCT patients diagnosed with IA	ASCT patients without IA	*p*-value‡
**Total No.**	**44**	**64**	
**Sex ratio male/female**	18/26	35/29	0.70
**Median age (IQR)**	47 (41–57)	51 (46–58)	0.26
**Hematological disease n (%)** *			
AMLMultiple myelomaCMLNHLALLAplastic anemiaCLLMDSOther	12 (27)8 (18)8 (18)7 (16)3 (7)1 (2)3 (7)2 (5)-	19 (30)11 (17)5 (8)11 (17)4 (6)4 (6)4 (6)4 (6)2 (3)	
**EORTC/MSG 2008 classification**			
ProvenProbable	539	--	
**Site of IA n (%)**			
Pulmonary	42 (95)	-	
Extra-pulmonary	2 (5)	-	
**Complications n (%)**			
Prolonged neutropenia†	15 (34)	29 (45)	0.32
GVHD	25 (57)	21 (32)	0.02
CMV IgG positive¶	14 (39)	34 (53)	0.21

**Legenda:** IA: invasive aspergillosis; IQR: interquartile range, ASCT: allogeneic stem cell transplantation, CMV: Cytomegalovirus, AML: acute myeloid leukemia, CML: chronic myeloid leukemia, NHL: non-Hodgekin's lymphoma, ALL: acute lymphocytic leukemia, CLL: chronic lymphocytic leukemia, MDS: myelodysplastic syndrome.

†: Prolonged neutropenia was defined as absolute neutrophil count <500 cells/mm^3^ for a period of more than 14 days.

¶: IgG positive prior to transplantation. GVHD: graft versus host disease;

‡: p-values were calculated by student-t test for continous- and Fishers exact test for binary data.

*: The distribution of hematological diseases was comparable between groups (Pearson Chi-Square test p = 0.85).

### Polymorphisms and genotyping

Polymorphisms were considered eligible for study if the SNP was previously reported to be associated with the occurrence of IA and had an expected allele frequency of ≥5% in the population. With regard to the focus of interest as pointed out in the introduction, two SNPs reported to influence IL-12p40 and IFN-γ production were additionally included (table 2). Blood- or bone marrow samples were used to isolate DNA. Genotyping of polymorphisms was performed by use of a Sequenom MassArray® platform according to the manufacturer's protocols (Sequenom, San Diego, USA). Multiplex assays were designed using Assay designer software (Sequenom). In brief, after PCR on 2.5 ng of DNA a primer extension reaction was performed to introduce mass-differences between alleles and, after removing salts by adding a resin, ∼15 nl of the product was spotted onto a target chip with 384 patches containing matrix. Mass differences were detected using a Bruker Autoflex MALDI-TOF mass spectrometer and genotypes were assigned real-time using Typer 3.1 software (Sequenom). Several samples representing the various genotypes were sequenced to confirm the genotyping results. As quality control, 10% of samples were genotyped in duplo; no inconsistencies were observed. Primer sequences are available upon request.

**Table 2:** Genetic polymorphisms in the innate immune system considered of potential influence on susceptibility to invasive aspergillosis.

Gene name	SNPdb id	Position nucleotide change	Reported effect	References
*IL1B*	rs16944	-511C>T	Negatively Influences IL-1β levels. A higher frequency of the *IL1B* -511TT genotype was found in patients with IA as compared to patients without IA.	Wilkinson et al. [31]Sainz et al. [32]
*IL10*	rs1800872	-592A>C	Promotor SNP, protective effect in conjunction with the −1082 and −819 *IL10* promotor polymorphisms	Seo et al. [33]
*IL10*	rs1800896	-1082G>A	Promotor SNP, conferring a diminished expression of the *IL10* gene and a subsequent protective effect with respect to IA	Sainz et al. [22]
*IL12B*	rs41292470	GC>CTCTAA	Promotor SNP, reported influence on response to tuberculosis; association with IA unknown	Sahiratmadja et al. [34]
*IFNG*	rs2430561	874T>A	Confers dimished production of IFN-γ, resulting in decreased activation of macrophages. Reported to influence cellular response to tuberculosis. Association with IA unknown.	Pravica et al. [35] Rossouw et al. [36]
*TLR1*	rs5743611	239G>C	Associated with IA in ASCT recipients	Kesh et al. [16]
*TLR1*	rs4833095	743A>G	Associated with IA in ASCT recipients when present in combination with the *TLR6* 745C>T polymorphism	Kesh et al. [16]
*TLR4*	rs4986791	1363C>T	Associated with IA when present in donor DNA of ASCT recipients	Bochud et al. [14]
*TLR4*	rs4986790	1063A>G	Associated with IA in ASCT when present in recipient DNA Associated with IA when present in donor DNA of ASCT recipients	Carvalho et al. [15] Bochud et al. [14]
*TLR6*	rs5743810	745C>T	Associated with IA in ASCT recipients when present in combination with the *TLR1* 743A>G polymorphism	Kesh et al. [16]

**Legenda:** IL denotes interleukin; TLR: toll-like receptor; IFN: interferon; ASCT: allogeneic stem cell transplantation; IA: invasive aspergillosis; SNPdb id: Single Nucleotide Polymorphism database identification number.

### Statistical analysis

Genotype- and allele frequencies were calculated and compared between groups by Pearson-chi-square and Fisher's exact tests. Odds ratio (OR) and 95% confidence interval (95%CI) were calculated for the presence (homozygous or heterozygous) or absence (homozygous wild type allele) of the selected SNPs. All polymorphisms were tested for the Hardy-Weinberg equilibrium. Due to the possibility that development of IA was influenced by SNPs in the donor DNA, genotype and allele frequencies were also compared between donors of the patients with IA and donors of control patients. Because of expected redundancy and complexity in the pathway to granulocyte and macrophage activation, the relevance of the combined presence of the selected polymorphisms was assessed in a contingency table. The outcomes were corrected for GVHD, CMV serostatus of recipient and donor, and for the duration of neutropenia. Bonferroni correction was not applied as the SNPs analyzed were each preselected based on clinical relevance [21]. The influence of a selected SNP on the course of disease (i.e., duration of neutropenia to day of diagnosis or time from IA diagnosis to death) was assessed by Kaplan-Meier analysis (log rank-test). The SPSS version 17.0 statistical software package for Windows was used for all calculations.

## Results

A total of 10 candidate polymorphisms, all acting within the type-1 cytokine loop to macrophage activation, were selected for analysis: five SNPs in three different *TLR* genes (−1,−4 and −6) and five SNPs in the *IL10*, *IL12B* and *IFNG* genes (table 2). The distribution of genotypes was consistent with the Hardy-Weinberg equilibrium except for the *IL12B* SNP. The *TLR4* 1063A>G and *TLR4* 1363C>T SNPs were in strong linkage disequilibrium, i.e. when the 1063A>G SNP was found, the 1363C>T was almost always also present. No significant difference in genotype or allele frequencies was found between patients with IA and control patients (data not shown). When comparing donor genotype and allele frequencies, the *TLR4* 1363C>T and *TLR4* 1063A>G SNPs were more frequently present in donors of patients with IA (table 3). The donor DNA contained the *TLR4* 1063A>G SNP in 9 of the 43 case patients (21%) and in 4 (7%) of the 61 control patients successfully genotyped for this polymorphism (OR 3.77 95%CI 1.08–13.2, p = 0.03). Following multivariate correction for GVHD, CMV serostatus and duration of neutropenia the adjusted OR was 3.76 (95% CI 0.90–15.8, p = 0.07). In addition, the allele frequency of the *IFNG* 874T>A polymorphism showed a trend towards association with IA when present in donors of patients with IA (OR 1.60 95%CI 0.91–2.79, p = 0.10).

**Table 3:** Genotype and allele frequencies of SNPs in *TLR*, *IL10, IL12* and *IFNG* genes in the donor DNA of patients who developed invasive aspergillosis after allogeneic stem cell transplantation.

gene	SNP	Distribution of Genotypes (mm/mM/MM)in Donors of ASCT recipients*		Allele frequency of the minor allele		*X^2^*	*p-value*	OR (95%CI)
		*cases*	*controls*	*cases*	*controls*			
***IL1B***	-511C>T	2/19/19	5/18/28	0.20	0.20	0.01	0.92	0.96 (0.48–1.96)
***IL10***	-592A>C	2/15/26	3/19/31	0.22	0.24	0.06	0.81	0.92 (0.47–1.81)
***IL10***	-1082G>A	5/24/14	13/26/15	0.40	0.48	1.44	0.23	0.70 (0.40–1.25)
***IL12B^∑^***	GC>CTCTAA	11/21/9	10/39/10	0.52	0.50	0.12	0.73	1.10 (0.63–1.94)
***IFNG***	874T>A	11/21/11	8/31/22	0.50	0.39	2.71	0.10	1.60 (0.91–2.79)
***TLR1***	239G>C	1/5/36	0/9/50	0.08	0.08	0.03	0.86	1.10 (0.39–3.08)
***TLR1***	743A>G	2/16/25	4/20/37	0.23	0.23	0.003	0.96	1.02 (0.53–1.96)
***TLR4***	1363C>T	1/7/34	0/5/56	0.11	0.04	3.43	0.06	2.81 (0.91–8.70)
***TLR4***	1063A>G	2/7/34	0/4/57	**0.13**	**0.03**	**6.82**	**0.01**	**4.33 (1.33–14.1)**
***TLR6***	745C>T	7/25/10	11/29/19	0.46	0.43	0.20	0.67	1.14 (0.65–2.00)

**Legenda:** IL denotes interleukin; TLR: toll-like receptor; IFN: interferon; ASCT: allogeneic stem cell transplantation; IA: invasive aspergillosis; *X* ***^2^***: chi-square test value; OR: odds ratio; 95%CI: 95% confidence interval. ∑: distribution of this genotype was not in Hardy-Weinberg equilibrium (p = 0.045).

*:Due to incidental failing of genotyping the No. of cases and controls are not equal for each SNP; m: minor allele; M:major allele.

Since our hypothesis was that susceptibility to IA by genetic mutations could be influenced by the interplay of both TLR and cytokine gene mutations, relevance of the combined presence of the selected polymorphisms was assessed in a contingency table (i.e. association of occurrence of IA with the presence of at least one minor allele in both genes in the interaction term). With respect to this analysis no significant associations with IA were found in the comparison of patients with IA versus control patients. However, a similar analysis performed for the genotypes of the donor samples revealed that paired combinations of the *TLR4* 1063A>G, *TLR*6 745C>T, or *IFNG* 874T>A SNPs correlated with occurrence of IA in the recipient (table 4). After multivariate adjustment for GVHD, CMV serostatus and neutropenia, only the association between the *TLR4* 1063A>G and *IFNG* 874T>A combination and IA remained statistically significant (p = 0.044). When using a forward conditional logistic regression model for assessment of strength of the association of individual or paired polymorphisms with IA, incorporating both the single presence of the minor SNP in the *TLR4*, *TLR6* and *IFNG* genes as well as their paired combinations, showed that the *TLR4* 1063A>G*/IFNG* 874T>A combination was most strongly linked with IA (p = 0.033).

**Table 4:** Final results of contingency table analysis for the association between the paired presence of TLR and cytokine polymorphisms in donors of ASCT recipients and development of invasive aspergillosis using all 10 polymorphisms.

Paired TLR - or cytokine SNPs	unadjusted OR	95% CI	*P^∫^*	adjusted* OR	95%CI	*p*
*TLR4* 1063A>G and *IFNG* 874T>A	5.74	1.13–29.1	0.035	6.09	1.05–35.5	0.044
*TLR6* 745C>T and *IFNG* 874T>A	2.15	0.96–4.80	0.064	1.80	0.33–4.32	0.189
*TLR4* 1063A>G and *TLR6* 745C>T	3.24	0.91–11.6	0.071	3.22	0.79–13.3	0.102

**Legenda:** *∫*: p-value calculated with Fisher's exact test. IL: interleukin; TLR: toll-like receptor; IFN: interferon.

*: Adjusted for presence of GVHD, CMV serostatus of donor and acceptor (either one or both CMV IgG+), and prolonged neutropenia (>14 days) by binary logistic regression. OR: odds ratio; 95%CI: 95% confidence interval.

Kaplan-Meier analysis did not reveal significant differences in time to development of IA between recipients or their donors bearing either only the wild-type or variant allele. There was no difference in survival (time to death following diagnosis of IA) consequent to having one or two minor alleles of the selected SNPs in either recipients or their donors.

## Discussion

We found that in this study cohort the *TLR4* 1063A>G polymorphism was associated with increased susceptibility to IA, when present in the donors DNA of ASCT recipients (alone or in combination with the *IFNG* 874T>A SNP). None of the cytokine polymorphisms alone were linked with occurrence of IA. The results of our investigations concur with the study by Bochud et al., in which the 1363C>T and 1063A>G polymorphisms in the *TLR4* gene were demonstrated to be associated with IA when present in donors of ASCT recipients [14]. In contrast, an increased risk for IA was previously reported for the 1063A>G SNP if present in the recipients DNA but not in the donor DNA [15]. The association between IA and the *TLR1* 239G>C SNP or between IA and the combination of the *TLR1* 743A>G and *TLR6* 745C>T SNPs as reported in a smaller study by Kesh et al. [16], was not confirmed by our data.

The *IFNG* 874T>A SNP was found to potentially add up to the risk conferred by two of the TLR polymorphisms. Although carriers of this genetic variation produce suboptimal levels of IFN-γ, putting them at increased risk for perhaps manifest tuberculosis, the isolated presence in either donor or recipient did not increase the risk for IA. Remarkably, SNPs that affect the production of IL-10, one of the most important broad-acting negative modulators of the TLR to IL-12 and IFN-γ macrophage-activating pathway, did not influence susceptibility to IA. Absence of IL-10 was demonstrated to cause increased survival of susceptible mice when exposed to *Aspergillus fumigatus* and in a prospective clinical study a tendency towards protection against IA was detected when the -1082 A/A-genotype was present [22].

As compared to other risk factors, the absolute risk conferred by relevant SNPs in PRR- and cytokine genes is likely to be limited, given the fact that individuals carrying these SNPs do not develop IA unless another immune deficiency is present. Moreover, our data indicate that even in hosts most at risk, the ability to maintain a response to IA is largely unaffected by the studied SNPs, underscoring the already expected redundancy inherent to the human antifungal defense [23]. Likely, specific patterns of genetic polymorphisms rather than a single genetic variation in TLRs or subsequent cytokine pathways that activate macrophages may be associated with IA in patients at risk. The observation of the association between the *TLR4* 1063A>G plus *IFNG* 874T>A SNP combination and IA fits such a hypothesis. However, probable associations of IA with conditional combinations of mutations may also attest to the complex immuno-pathogenesis of invasive aspergillosis. As a consequence of neutropenia, the role of key components within the innate immune response (e.g. lung macrophages phagocytosing and eliminating Aspergillus conidia) could be more prominent in the remaining defense against invasive fungal infection and thus facilitate linkage to TLR- in combination with cytokine SNPs. Assuming that the studied SNPs have an effect on the functioning of the innate immune system, different SNPs may also be working at different time points to modulate resistance to IA and eventually constitute the hosts genetic signature of susceptibility.

Of note, recent publications suggest that components of the innate immunity, including TLRs, may be linked to the pathogenesis of hematological malignancies [24]–[27]. Although in our study a control group with a comparable distribution of hematological malignancies was used, this intriguing loop may complicate the interpretation of studies in this field. Furthermore, the study has its limitations, e.g. due to a retrospective design and size of the study cohort. This prevents the incorporation of a larger amount of variables to correct for in the multivariate analyses (e.g. duration of follow-up post transplantation). However, two studies that explored the role of TLR SNPs and risk for IA included a comparable or even smaller number of patients [15], [16]. Of note, rather than performing a genome-wide analysis, or testing a random collection of immune genes, we chose to investigate the association of IA with pre-set polymorphisms in candidate genes involved in type-1 cytokine loop to macrophage activation. This ameliorates implications with regard to the concept of multiple testing [21], but by some a significance level of 0.05 may still be regarded too liberal. Currently an ongoing discussion about the necessity of *p-*value adjustment in exploratory epidemiological studies still evolves and with all relevant data reported, final judgment is left to the reader [28], [29]. Furthermore, due to the rapidly evolving research field, producing newly found candidate SNPs like the Dectin-1 Y238X polymorphism, investigations can hardly ever be complete [30].

The overall impact of the reported *TLR4* 1063A>G and *IFNG* 874T>A SNPs on the risk of IA should be interpreted with care. Relative risk associations of genetic variations in the case of IA do not stand alone but likely are influenced again by other components in the host's defense. Due to study limitations, the outcomes were corrected only for known important clinical risk factors, but not for other variables. As discussed above, the observations of this study may be accounted for by both a system of redundancy in the innate immune system as well as by the complex biological interaction between the immunocompromised host and the invading fungus. At present, the findings do not extent to the bedside yet, e.g., by providing guidance for individualized prophylaxis or early intervention. However, by further unravelling the interplay between the innate host defence and *Aspergillus sp.* through experimental and clinical investigations, increased comprehension of the underlying immuno-pathogenetic processes may, in time, translate into insights directly relevant to clinical practice.

## References

[1] Neofytos D, Horn D, Anaissie E, Steinbach W, Olyaei A (2009). Epidemiology and outcome of invasive fungal infection in adult hematopoietic stem cell transplant recipients: analysis of Multicenter Prospective Antifungal Therapy (PATH) Alliance registry.. Clin Infect Dis.

[2] Marr KA, Carter RA, Boeckh M, Martin P, Corey L (2002). Invasive aspergillosis in allogeneic stem cell transplant recipients: changes in epidemiology and risk factors.. Blood.

[3] Upton A, Kirby KA, Carpenter P, Boeckh M, Marr KA (2007). Invasive aspergillosis following hematopoietic cell transplantation: outcomes and prognostic factors associated with mortality.. Clin Infect Dis.

[4] Pagano L, Caira M, Candoni A, Offidani M, Martino B (2010). Invasive aspergillosis in patients with acute myeloid leukemia: a SEIFEM-2008 registry study.. Haematologica.

[5] Avery RK (2009). Aspergillosis in hematopoietic stem cell transplant recipients: risk factors, prophylaxis, and treatment.. Curr Infect Dis Rep.

[6] Bjorklund A, Aschan J, Labopin M, Remberger M, Ringden O (2007). Risk factors for fatal infectious complications developing late after allogeneic stem cell transplantation.. Bone Marrow Transplant.

[7] Luther K, Rohde M, Sturm K, Kotz A, Heesemann J (2008). Characterisation of the phagocytic uptake of Aspergillus fumigatus conidia by macrophages.. Microbes Infect.

[8] Shoham S, Levitz SM (2005). The immune response to fungal infections.. Br J Haematol.

[9] Meier A, Kirschning CJ, Nikolaus T, Wagner H, Heesemann J (2003). Toll-like receptor (TLR) 2 and TLR4 are essential for Aspergillus-induced activation of murine macrophages.. Cell Microbiol.

[10] Netea MG, Warris A, Van der Meer JW, Fenton MJ, Verver-Janssen TJ (2003). Aspergillus fumigatus evades immune recognition during germination through loss of toll-like receptor-4-mediated signal transduction.. J Infect Dis.

[11] Netea MG, Van der Graaf C, Van der Meer JW, Kullberg BJ (2004). Recognition of fungal pathogens by Toll-like receptors.. Eur J Clin Microbiol Infect Dis.

[12] Takeda K, Akira S (2005). Toll-like receptors in innate immunity.. Int Immunol.

[13] Chai LY, Kullberg BJ, Vonk AG, Warris A, Cambi A (2009). Modulation of Toll-like receptor 2 (TLR2) and TLR4 responses by Aspergillus fumigatus.. Infect Immun.

[14] Bochud PY, Chien JW, Marr KA, Leisenring WM, Upton A (2008). Toll-like receptor 4 polymorphisms and aspergillosis in stem-cell transplantation.. N Engl J Med.

[15] Carvalho A, Pasqualotto AC, Pitzurra L, Romani L, Denning DW (2008). Polymorphisms in Toll-Like Receptor Genes and Susceptibility to Pulmonary Aspergillosis.. J Infect Dis.

[16] Kesh S, Mensah NY, Peterlongo P, Jaffe D, Hsu K (2005). TLR1 and TLR6 polymorphisms are associated with susceptibility to invasive aspergillosis after allogeneic stem cell transplantation.. Ann N Y Acad Sci.

[17] Lasker MV, Nair SK (2006). Intracellular TLR signaling: a structural perspective on human disease.. J Immunol.

[18] Brieland JK, Jackson C, Menzel F, Loebenberg D, Cacciapuoti A (2001). Cytokine networking in lungs of immunocompetent mice in response to inhaled Aspergillus fumigatus.. Infect Immun.

[19] Cenci E, Mencacci A, Fe d' C, Del Sero G, Mosci P (1998). Cytokine- and T helper-dependent lung mucosal immunity in mice with invasive pulmonary aspergillosis.. J Infect Dis.

[20] de Pauw B, Walsh TJ, Donnelly JP, Stevens DA, Edwards JE (2008). Revised definitions of invasive fungal disease from the European Organization for Research and Treatment of Cancer/Invasive Fungal Infections Cooperative Group and the National Institute of Allergy and Infectious Diseases Mycoses Study Group (EORTC/MSG) Consensus Group.. Clin Infect Dis.

[21] Perneger TV (1998). What's wrong with Bonferroni adjustments.. BMJ.

[22] Sainz J, Hassan L, Perez E, Romero A, Moratalla A (2007). Interleukin-10 promoter polymorphism as risk factor to develop invasive pulmonary aspergillosis.. Immunol Lett.

[23] Chai LY, Netea MG, Vonk AG, Kullberg BJ (2009). Fungal strategies for overcoming host innate immune response.. Med Mycol.

[24] Abdi J, Engels F, Garssen J, Redegeld F (2011). The role of Toll-like receptor mediated signalling in the pathogenesis of multiple myeloma.. Crit Rev Oncol Hematol.

[25] Dickinson AM, Pearce KF, Norden J, O'Brien SG, Holler E (2010). Impact of genomic risk factors on outcome after hematopoietic stem cell transplantation for patients with chronic myeloid leukemia.. Haematologica.

[26] Liang XS, Caporaso N, McMaster ML, Ng D, Landgren O (2009). Common genetic variants in candidate genes and risk of familial lymphoid malignancies.. Br J Haematol.

[27] Nieters A, Beckmann L, Deeg E, Becker N (2006). Gene polymorphisms in Toll-like receptors, interleukin-10, and interleukin-10 receptor alpha and lymphoma risk.. Genes Immun.

[28] Feise RJ (2002). Do multiple outcome measures require p-value adjustment?. BMC Med Res Methodol.

[29] Rothman KJ (1990). No adjustments are needed for multiple comparisons.. Epidemiology.

[30] Plantinga TS, van der Welden WJ, Ferwerda B, van Spriel AB, Adema G (2009). Early stop polymorphism in human DECTIN-1 is associated with increased candida colonization in hematopoietic stem cell transplant recipients.. Clin Infect Dis.

[31] Wilkinson RJ, Patel P, Llewelyn M, Hirsch CS, Pasvol G (1999). Influence of polymorphism in the genes for the interleukin (IL)-1 receptor antagonist and IL-1beta on tuberculosis.. J Exp Med.

[32] Sainz J, Perez E, Gomez-Lopera S, Jurado M (2008). IL1 gene cluster polymorphisms and its haplotypes may predict the risk to develop invasive pulmonary aspergillosis and modulate C-reactive protein level.. J Clin Immunol.

[33] Seo KW, Kim DH, Sohn SK, Lee NY, Chang HH (2005). Protective role of interleukin-10 promoter gene polymorphism in the pathogenesis of invasive pulmonary aspergillosis after allogeneic stem cell transplantation.. Bone Marrow Transplant.

[34] Sahiratmadja E, Baak-Pablo R, de Visser AW, Alisjahbana B, Adnan I, van Crevel R (2007). Association of polymorphisms in IL-12/IFN-gamma pathway genes with susceptibility to pulmonary tuberculosis in Indonesia.. Tuberculosis (Edinb).

[35] Pravica V, Perrey C, Stevens A, Lee JH, Hutchinson IV (2000). A single nucleotide polymorphism in the first intron of the human IFN-gamma gene: absolute correlation with a polymorphic CA microsatellite marker of high IFN-gamma production.. Hum Immunol.

[36] Rossouw M, Nel HJ, Cooke GS, van Helden PD, Hoal EG (2003). Association between tuberculosis and a polymorphic NFkappaB binding site in the interferon gamma gene.. Lancet.

